# Atlanta Society of Medicine

**Published:** 1895-07

**Authors:** 


### SOCIETY REPORTS.

---

### ATLANTA SOCIETY OF MEDICINE.

ATLANTA, May 21, 1895.

*The President, Dr. C. D. Hurt, in the Chair.*

The regular order of the meeting was the reading of papers by DR. J. C. JOHNSON and DR. BERNARD WOLFF.

DR. JOHNSON'S paper was entitled:

### "FEVER AS A DISEASE AND AS A SYMPTOM OF DISEASE."

Fever always denotes disease. It is a symptom of perturbated activity. The animal body has a certain caloric habit and capacity of heat production and heat elimination, and these are usually so evenly adjusted, in health, that the bodily temperature remains fixed even under differing external conditions of temperature. Certain influences may disturb this even balance of heat production and heat dissipation. If this be a disease the fever is called essential or idiopathic; or the fever may be one of the evidences of some well-defined disease; it is then symptomatic only. The source of the heat in fever cannot be due to increased combustion, the union of oxygen with carbon and other elements, since in debilitating diseases, such as typhoid, pneumonia, and phthisis, where the number and size of the red blood cells are lessened and the vital capacity of the lungs much diminished, the fever often runs continuously high; also, elevations of temperature are not infrequently found *post mortem*, due probably to the surrender of latent heat from the tissues. The theory that fever is caused by the irritation of certain heat centers, supposed to be situated near the optic thalmi and corpora striata, is not universally accepted.

Let us look for a moment at fever as a condition. The surface of the body is hot and dry, the face is flushed, the pulse is rapid and strong or feeble as the patient is sthenic or asthenic. The appetite is impaired, digestion is imperfect, the secretions and excretions scanty, the nervous system is variously disturbed, there is pain, perhaps delirium, the muscles are sore and weak. Are these associate symptoms with the fever dependent upon a common cause? Are they the result of the fever, or do they bear a part in its production? Remedies which lessen secretion do not, *per se*, elevate the temperature, neither do they lower it, save by removing or mitigating the condition which bears a direct causative relation to it.

It is true that there are agents which will at the same time reduce temperature and relieve pain, but that they do so without direct or material effect upon the cause of the fever, is proven by the fact that both return when the action of the medicine is exhausted or withdrawn. Neither do such agents shorten the duration of the attack. Opium will reduce fever due to irritation by obtunding the reflex sensibilities, but it is ineffective upon others.

In the febrile disturbances it becomes a matter of considerable interest to determine in what tissues the changes which are taking place occur, and what is the nature of these changes. The blood has been suggested by some as the medium through which the poisons have been absorbed and are operating. This does not explain the *modus operandi*, for "a poison is such only by its stimulant, depressant, irritant, or alterative effects." This does not explain the existence of febrile disturbance, for the poisonous element may be present and all symptoms of this condition absent.

Fever is due largely to an interference with trophic activities. The pathology of essential fever involves chiefly the sympathetic system, through the medium of the blood, which undergoes changes dependent upon the action of germs or poisons on its plasma, thereby generating heat. There is a lack of potential energy, and each organ is called upon to compensate for this deficiency. No fixed rule can be laid down for treatment of fever; its cause, degree, associate symptoms and conditions alone determine what is to be done in each case. In inflammatory fever, simple or traumatic remedies which control the perverted vascular system are indicated, viz: veratrum, opium, quinine, bloodletting, counter-irritation. It is different in essential fevers: these are self-limited and cannot be cut short; the temperature may be lowered to normal, but will not remain there without the continuous use of remedies. By an attempt to keep it there we may or may not conserve the strength of the patient.

Nature's first effort is to expel the cause. In the eruptive fevers this is accomplished chiefly through the skin. In typhoid fever the evacuations from the bowels carry off the bacteria, largely producing conservative diarrhea.

It is expedient to have regular evacuations of bowels, and this is most safely accomplished by hot enemata. A mild purge sometimes does well, but should be judiciously used. Nature's method of reducing fever is by evaporation by skin cooling off surface, exhalation from lungs, by urine, discharges from bowels. Strict attention should be given all three functions, and assistance by appropriate remedies used when necessary. Antipyretics which are at the same time stimulants are worthy of consideration. When the latter agents are carelessly or injudicously used they are capable of serious harm. The bath has stood the test of experience better as an agent for the reduction of temperature.

A reduction is not safe that does not conserve the vital force. Mercury may do good in typhoid fever and diphtheria if timely given and in proper doses.

Nutritious diet which taxes digestive apparatus least and leaves smallest residue should be used. Liberal use of water is grateful and beneficial. Muscular repose and complacency of mind are essential factors. In the main, our efforts should be directed to the preservation of all the organs and their functions in as near the normal state as possible, and inviting a speedy convalescence.

Dr. Wolff read a paper on

"THE NATURE AND TREATMENT OF ACNE."

He said that this disease was formerly regarded as a cutaneous manifestation of some systemic vice. Attempts to prove its purely local origin and to determine the proper micro-organism of acne have not been successful, and clinical evidence and observation are against such an explanation; so that we are little nearer the exact truth than before.

The disease consists in an inflammatory condition of the sebaceous glands and their excretory ducts, or in addition, an inflammation around the glands. The hyper-secretion becomes blocked and a comedone forms; this acts as a foreign body, and a pustule is the result. The pustule finally ruptures and the contents, including the plug of sebum, is discharged. The inflammation subsides, and the sebaceous gland returns to its natural condition or is obliterated. Although the disease commonly begins about the age of puberty, at the time when the sexual development is influencing the whole system, this condition is not *per se* capable of determining the appearance of acne, and the majority of mankind pass through this epoch without any, or very slight, evidence of the disease. Occupation has some influence. Workers in tar are liable to it, and those employed in candy factories and stores. Disorders of the digestive organs exert a reflex causative influence; also indiscretions in diet, overeating, drinking malt liquors, sweet wine, etc., or any reflex disturbance that serves to lower the tone of the system in an individual possessing a proclivity to that disease.

Acne, except in mild cases, cannot be cured outright, but it can be more or less controlled. The treatment is general and local. Internal treatment is entirely symptomatic. Whatever depressing influences can be found must be combated by appropriate means. Bowels should be kept open. Diet should be simple and free from anything to cause flatulence or fermentative dyspepsia. The local treatment must be mainly relied on. My practice is to curette the face thoroughly, tear off the tops of the pustules, liberate the contents and drag out the comedones. The larger pustules should be incised with the acne lancet, and contents expressed or extracted. A sulphur lotion may then be used. Such as:

℞ Zinci sulphat.<br>

Potass. sulphid. } āā ........................................ ʒ i.<br>

Sulph. precipitat.<br>

Aquæ rosæ........................................................... ℥ ii.

This should be applied every night after washing the face in tepid water to which borax may have been added. If this does not remove the eruption in several weeks the following salve is recommended to be used at night (during the day the compound stearate of zinc may be used):

| | | |
|---|---|---|
| ℞ | Hydrarg. bichloride | gr. i–ij. |
| | Resorcin. | ʒ ss–i. |
| | Aq. lauro-cerasi | ʒ ij. |
| | Farinæ | ʒ ij. |
| | Lanolin, ad | ℥ i. |

This should be used for some time.

In acne indurata the deep-seated lesions should be incised, the contents removed, and the ointment of resorcin, ichthyol and sulphur (each one dram to the ounce of lanolin) used. Curetting every week or ten days will be of benefit.

All remedies used in the local treatment of acne are antiseptics, the choice being determined by the degree of antiseptic influence desired. Soap and water should be used throughout the whole course of treatment for the sake of cleanliness. Carbolic soap is alone sufficient in the treatment of acne of the back. Rarely is anything more than cleanliness and some antiseptic necessary to relieve acne of the back.

---

### THE TREATMENT OF DIPHTHERIA.

While antitoxin is on probation the other methods of treating diphtheria should neither be overlooked nor abandoned. It is not yet proven that antitoxin is the long-wished-for specific. Dr. L. Emmet Holt, of New York, thus outlines the proper management of this disease:

1. Germicidal treatment, preferable by the use of strong hydrochloric acid, used early to be effectual; especially valuable in cases beginning on the tonsils.

2. Local cleanliness by the use of a weak antiseptic solution in the pharynx.

3. Nasal spraying with the same solution in every case where there is nasal discharge.

4. Alcoholic stimulants begun as soon as the first systemic effects of the poison are seen, and in very severe cases pushed to the point of tolerance.

5. Calomel fumigations as soon as laryngeal symptoms appear.

6. Intubation in laryngeal cases not relieved by fumigations.

